# Comparison of qSOFA Score, SIRS Criteria, and SOFA Score as predictors of mortality in patients with sepsis

**DOI:** 10.4314/gmj.v56i3.9

**Published:** 2022-09

**Authors:** A M Khan, Shaikh M Aslam

**Affiliations:** 1 Department of General Medicine, M. S. Ramaiah Medical College, Bangalore, Karnataka, India; 2 Department of General Medicine, M. S. Ramaiah Medical College, Bangalore, Karnataka, India

**Keywords:** Sepsis, Systemic inflammatory response syndrome, Mortality, Prognosis, Intensive care units

## Abstract

**Objectives:**

Early diagnosis and treatment of sepsis are associated with a better outcome. With the change in the definition of sepsis, SOFA score and qSOFA score (heart rate, systolic blood pressure and Glasgow coma scale) were introduced and SIRS criteria were removed. This study compared the qSOFA score, SIRS criteria and SOFA score as predictors of mortality in patients with sepsis.

**Design:**

Prospective observational study.

**Setting:**

Department of General Medicine of a tertiary hospital.

**Participants:**

The study included 116 patients.

**Interventions:**

SOFA scores (range, 0 [best] to 24 [worst] points), SIRS status (range, 0 [best] to 4 [worst] criteria), and qSOFA scores (range, 0 [best] to 3 [worst] points) were calculated using physiological and laboratory parameters recorded within the first 24 hours of ICU admission.

**Main outcome measures:**

SOFA, qSOFA, and SIRS scores were calculated and measured using physiological and laboratory parameters. Patients were followed till mortality (non-survivors) or discharge from the hospital (survivors). Data were analysed using software SPSS version 20.

**Results:**

54 (46.6%) of included patients died. Higher SOFA, qSOFA, and SIRS scores; tachycardia; hypotension; hypoxemia; basophilia; hypoproteinemia; hypoalbuminemia; and need for inotropic support and mechanical ventilation significantly associated with increased mortality. The area under the receiver operating curve for qSOFA ≥2 (0.678; p=0.001) and SOFA (0.74; p=0.000) were comparable and significant, whereas SIRS ≥2 (0.580, p=0.139) was not statistically significant.

**Conclusions:**

A qSOFA score of greater than 2 is comparable to SOFA and is better than SIRS score greater than 2 for predicting mortality.

**Funding:**

None indicated

## Introduction

Sepsis refers to a potentially life-threatening condition caused by the body's extreme response to an infection, which can rapidly lead to tissue damage, organ failure, and death.^1^ It has an annual global incidence of 31.5 million cases, of which 19.4 million are cases of severe sepsis, resulting in 5.3 million deaths annually.^2^

Earlier, sepsis was defined as the combination of infection and systemic inflammatory response syndrome (SIRS).^3^ However, SIRS criteria are overly sensitive and insufficiently specific in identifying infected patients at risk for a complicated course.^4^ The Third International Consensus Definitions for Sepsis and Septic Shock (Sepsis-3) Task Force recently redefined sepsis as a life-threatening organ dysfunction caused by a dysregulated host response to infection.^5^ Organ dysfunction is characterised by the acute increase of at least two points in the Sequential Organ Failure Assessment (SOFA) score.^5^

Since SOFA requires laboratory testing and is and is rarely performed outside the intensive care unit (ICU), the Sepsis-3 Task Force introduced quick SOFA (qSOFA).^5^ qSOFA is a simple scoring system that can be repeatedly assessed by the bedside with ease. qSOFA consists of three clinical elements, namely hypotension, tachypnea, and altered consciousness.^5^ As per the literature, it has higher accuracy than SIRS score in predicting mortality in patients with suspected sepsis outside the ICU.^6^

Increased specificity of qSOFA over SIRS score for predicting poor prognosis may come at the expense of lower sensitivity, leading to delays in the initiation of treatment. There are conflicting reports, with some claiming superiority of SIRS over qSOFA, while others claim the opposite.^6^–^9^ Correct identification of mortality predictors would help timely detection and correction of any potentially fatal deterioration in patient condition and accordingly mobilise ICU resources.^10^ Hence, the present research aimed to compare SOFA score, qSOFA score, and SIRS criteria as predictors of mortality in patients with sepsis.

## Methods

This hospital-based prospective observational study was conducted at the Department of General Medicine of a tertiary hospital, from October 2017 to September 2019, after obtaining ethical clearance from the Institutional Ethics Committee of Ramaiah Medical College (SS-1/EC/025/2017).

The study included patients aged >18 years who were diagnosed at the time of admission to the hospital with sepsis as per the Third International Consensus Definitions for Sepsis and Septic Shock (Sepsis-3), after obtaining written informed consent from them or their attendees.^5^ All sedated patients were excluded from the study. Detailed clinical history and results of relevant investigations were recorded. This included age, gender, associated co-morbidities, cause for sepsis, microbial culture, vital parameters, and haematological investigations. SOFA scores (range, 0 [best] to 24 [worst] points),^5^,^11^ SIRS status (range, 0 [best] to 4 [worst] criteria),^3^ and qSOFA scores (range, 0 [best] to 3 [worst] points)^5^,^12^ was calculated using physiological and laboratory parameters recorded within the first 24 hours of ICU admission. The patients' need for inotropic support and mechanical ventilation was also noted. Patients were followed up till the end point i.e., either mortality (non-survivors) or discharge from the hospital (survivors).

### Statistical analysis

The sample size was calculated based on the research conducted by Raith et al wherein the area under the receiver operating curve (AUROC) for qSOFA score for predicting mortality was 60%.^6^ Accordingly, expecting similar results, for a relative precision of 15% and desired confidence level of 95% (5% alpha error), a sample size of 114 cases was required.

Data were compiled and analysed using the statistical software SPSS version 20. Descriptive and inferential statistical analyses were conducted in the present study. Measurements of continuous variables were expressed as mean standard deviation (minimum-maximum), and those of categorical variables were presented in number (%) format. Student t-tests assessed the significance of study parameters on a continuous scale between two groups (inter-group analysis) on metric parameters. Chi-square/Fisher's Exact test assessed the significance of study parameters on a categorical scale between two or more groups non-parametric setting for qualitative data analysis. ROC was employed to compare the qSOFA, SIRS, and SOFA scores. ROC curves were compared using the empirical (nonparametric) methods. The Youden Index was used to identify the best cut-off value. Significance was determined at a 5% level of significance.

## Results

The study consisted of 116 sepsis patients and a M: F = 1:1 with a mean age of 53.25 ± 18.39 years, belonging mostly to the 40–60 years age group. Of these, 62 (53.4%) patients survived, and 54 (46.6%) died. Type 2 diabetes mellitus (35.3%) and hypertension (28.4%) were the most common comorbid conditions. Bronchopneumonia (44.8%) was the most common cause of sepsis, followed by urosepsis (22.4%). Table 1 presents the frequency distribution and intergroup comparison of the various parameters. No significant difference in age and gender distribution was observed between survivors and deceased patients. Culture positivity was seen in 44 (37.9%) of the patients. Among non-survivors, blood, urine, and sputum culture were positive in 9 (16.7%), 8 (14.8%), and 4 (7.4%) patients, respectively. E. coli was the most common organism isolated from blood (6%), urine (11.2 %), and sputum (1.8%). Requirement of inotropic support and mechanical ventilation was seen in 61 (52.6%) and 69 (59.5%) patients, respectively, out of which 38 (62.29%) and 49 (71.01%) patients, respectively, died. Compared to the survivors, the non-survivor group had a significantly higher proportion of patients placed on inotropic support (p=0.001) and mechanical ventilation (p=0.001). This implies that requirements for inotropic support and mechanical ventilation were significantly associated with increased mortality.55 patients (47.41%) had sepsis, and 61 (52.59%) were in septic shock. Out of the 54 patients who died, 16 patients (29.63%) had sepsis, and 38 (70.37%%) of the patients had septic shock. Mortality in the septic shock group was higher when compared to patients in the sepsis group (P=0.0003).

**Table 1:** Frequency distribution and intergroup comparison of the various parameters

Parameter		Non-survivors	Survivors	Total	p-value
**No. of patients**		54 (46.6%)	62 (53.4%)	116 (100%)	-
**Gender**	Male	28 (51.9%)	30 (48.4%)	58 (50%)	0.71
	Female	26 (48.1%)	32 (51.6%)	58 (50%)	
**Co-morbidities**	Diabetes	15 (27.8%)	26 (41.9%)	41 (35.3%)	0.11
	Hypertension	15 (27.8%)	18 (29.0%)	33 (28.4%)	0.88
	IHD	5 (9.3%)	6 (9.7%)	11 (9.4%)	0.93
	CVD	3 (5.6%)	1 (1.6%)	8 (6.8%)	0.24
	Hypothyroidism	2 (3.7%)	6 (9.7%)	3 (2.5%)	0.205
	Bronchial Asthma	1 (1.9%)	2 (3.2%)	3 (2.5%)	0.64
**Cause for sepsis**	Bronchopneumonia	26 (48.1%)	26 (41.9%)	52 (44.8%)	-
	Urosepsis	7 (12.9%)	19 (30.6%)	26 (22.4%)	
	CNS	3 (5.5%)	12 (19.3%)	15 (12.9%)	
	GI and Hepatobiliary	5 (9.2%)	3 (4.8%)	8 (6.8%)	
	Miscellaneous	13 (24.07%)	2 (3.2%)	15 (12.9%)	
**Culture**	Positive	22 (40.7%)	22(35.5%)	44(37.9%)	0.56
**Blood culture**	Positive	9 (16.7 %)	7 (11.3 %)	16 (13.8 %)	-
	Negative	45 (83.3 %)	55 (88.7 %)	100 (86.2%)	
**Organisms isolated** **from blood culture**	*E. coli*	6(11.1 %)	1(1.6 %)	7 (6.0 %)	-
	*P. aeruginosa*	1(1.8 %)	2 (3.2 %)	3 (2.5 %)	
	*Enterococcus*	0	2 (3.2 %)	2 (1.7 %)	
	*CNSA*	0	2 (3.2 %)	2 (1.7 %)	
	*MSSA*	2(3.7 %)	0	2 (1.7 %)	
**Urine culture**	Positive	8 (14.8%)	8 (12.9 %)	16 (13.8 %)	-
	Negative	46 (87.1%)	54 (85.2 %)	100 (86.2%)	
**Organisms isolated** **from urine culture**	*E. Coli*	5(14.8 %)	8(12.9 %)	13 (11.2 %)	-
	*C. ferundii*	0	1 (1.6 %)	1 (0.86 %)	
	*Enterococcus*	2(3.7 %)	0	2 (1.7 %)	
**Sputum culture**	Positive	4 (7.4 %)	3 (4.8 %)	7 (6 %)	-
	Negative	50 (92.6 %)	59 (95.2 %)	109 (94 %)	
**Organisms isolated** **from sputum culture**	*E. Coli*	1 (1.8 %)	1 (1.6 %)	13 (11.2 %)	-
	*P. aeruginosa*	1 (1.8 %)	1 (1.6 %)	1 (0.86 %)	
	*Acinetobacter*	1 (1.8 %)	1 (1.6 %)	2 (1.7 %)	
	*Klebsiella*	1 (1.8 %)	0	1(0.8 %)	
**Inotropic support**	Yes	38 (62.29%)	23 (37.70%)	61 (52.6%)	0.001*
**Mechanical ventilation**	Yes	49 (71.01%)	20 (28.98%)	69 (59.5%)	0.001*
**Group**	Sepsis	16 (29.63%)	39 (62.9%)	55 (47.41%)	0.0003*
	Septic Shock	38 (70.37%)	23 (37.1%)	61 (52.59%)	

Abbreviations: *Significant at 5% level of significance; C. ferundii = Citrobacter ferundii; CNS = Central nervous system; CNSA = Coagulase-negative Staphylococcus aureus; CVD = Cardiovascular disease; E. coli = Escherichia coli, GI = Gastrointestinal; IHD = Ischemic heart disease; MSSA = Methicillin-resistant Staphylococcus aureus; P. aeruginosa = Pseudomonas aeruginosa

Table 2 depicts the mean values and intergroup comparison of the clinical and laboratory parameters. Tachycardia was observed in all patients, with a mean heart rate of 109.75 ± 18.99 beats per minute (bpm). Anaemia, leukocytosis, and neutrophilia were the common abnormalities noted on the complete blood count. Hyperbilirubinemia, hypoalbuminemia, and transaminitis were common abnormalities noted on liver function tests.

**Table 2:** Mean values and intergroup comparison of the clinical and laboratory parameters

Parameter		Non-survivors	Survivors	Total	p-value
**Mean age (years)**		52.03 ± 19.09	54.32 ±17.84	53.25 ± 18.39	0.507
**Clinical parameters**	Mean PR (bpm)	114.46 ± 17.17	105.64 ± 19.66	109.75 ±18.99	0.01*
	Median PR (bpm)	113	108	110	0.01*
	Mean SBP (mmHg)	101.29 ± 27.89	112.19 ± 32.96	107.12 ± 31.06	0.05*
	Median SBP (mmHg)	90	110	100	0.05*
	Mean DBP (mmHg)	60.703 ± 17.85	70.58 ± 19.607	65.98 ± 19.37	0.006*
	Median DBP (mmHg)	60	70	60	0.006*
	Respiratory rate (cpm)	29.92 ± 5.28	28.29 ± 10.45	29.05 ± 8.457	0.3
	Temperature (°F)	99.71 ± 1.80	99.57 ± 1.81	99.63 ± 1.80	0.67
	Oxygen saturation (%)	83.85 ± 7.24	89.32 ± 8.12	86.77 ± 8.16	0.000*
**Complete blood** **count**	Hemoglobin (g/dL)	11.47 ± 2.91	11.29 ± 2.33	11.379 ± 2.61	0.71
	Total Count (cells/mm^3^)	13063.01 ± 7779.86	15961.77 ± 8651.002	14612.35 ± 8348.72	0.06
	Neutrophils (%)	80.79 ± 15.62	80.26 ± 14.67	80.514 ± 15.06	0.85
	Lymphocytes (%)	11.35 ± 8.84	12.306 ± 9.06	11.86 ± 8.93	0.57
	Eosinophils (%)	0.84 ± 2.18	0.74 ± 1.54	0.793 ± 1.86	0.76
	Basophils (%)	0.42 ± 0.85	0.18 ± 0.24	0.29 ± 0.619	0.04*
	Monocytes (%)	4.68 ± 2.53	4.88 ± 2.48	4.78 ± 2.496	0.67
	Platelets (lakhs/mm^3^)	1.67 ± 1.36	1.94 ± 1.41	1.82 ± 1.39	0.29
**Liver function** **tests**	Total Bilirubin (mg/dL)	2.43 ± 3.67	2.307 ± 4.69	2.36 ± 4.23	0.86
	Direct Bilirubin (mg/dL)	1.96 ± 3.29	1.909 ± 4.67	1.93 ± 4.07	0.93
	Total protein (g/dL)	5.73 ± 1.11	6.15 ± 1.24	5.95 ± 1.19	0.05*
	Albumin (g/dL)	2.83 ± 0.706	3.104 ± 0.66	2.97 ± 0.69	0.03*
	A/G Ratio	1.03 ± 0.32	1.21 ± 0.906	1.12 ± 0.701	0.17
	AST (IU/L)	226.74 ± 946.53	114.41 ± 386.62	166.70 ± 703.81	0.39
	ALT (IU/L)	118.79 ± 389.6	80.09 ± 333.55	98.11 ± 359.64	0.56
	ALP (IU/L)	188.72 ± 293.22	139.45 ± 112.07	162.38 ± 276.55	0.22
	GGT (IU/L)	97.27 ± 87.63	76.96 ± 78.11	86.42 ± 82.94	0.19
**Renal function** **tests**	BUN (mg/dL)	30.04 ± 19.74	28.02 ± 24.26	28.96 ± 22.20	0.66
	Creatinine (mg/dL)	1.79 ± 1.13	1.69 ± 1.34	1.74 ± 1.24	0.11
	Uric acid (mg/dL)	6.73 ± 3.33	5.81 ± 2.93	6.24 ± 3.14	0.22

Abbreviations: *Significant at 5% level of significance; A/G = Albumin/Globulin; ALP = Alkaline phosphatase; ALT = Alanine transaminase, AST = Aspartate transaminase; bpm = beats per minute; BUN = Blood urea nitrogen; cpm = counts per minute; DBP = Diastolic blood pressure; GGT = Gamma-glutamyl transferase; PR = pulse rate; SBP = Systolic blood pressure

Elevated mean creatinine level was a common abnormality noted on the renal function test. Compared to the survivors, the non-survivor group had a significantly higher mean pulse rate (PR) (p=0.01) and basophil count (p=0.04) and significantly lower SBP (p=0.05), diastolic blood pressure (DBP) (p=0.006), oxygen saturation (p=0.000), total protein (p=0.05), and albumin level (p=0.03). This implies that tachycardia, hypotension, hypoxemia, basophilia, hypoproteinemia, and hypoalbuminemia were significantly associated with an increase in mortality.

Table 3 summarises the intergroup comparison of SOFA, qSOFA, and SIRS scores. The mean SOFA score was significantly higher in non-survivors (9.09 ± 4.21) compared to that in survivors (5.75 ± 2.906) (p=0.001).

**Table 3:** Intergroup comparison of SOFA, qSOFA, and SIRS scores

Parameter		Non	Survivors	Total	p-value
Mean SOFA score		9.09 ± 4.21	5.75 ± 2.906	-	0.001*
Median SOFA score		8.5 (6, 12)	5 (3.25, 7)	7 (4, 9)	0.001*
SIRS criteria	1	1 (11.11%)	8 (88.88 %)	9 (7.8%)	0.04*
	2	18 (50 %)	18 (50%)	36 (31.0%)	
	3	28 (45.16%)	34 (54.83%)	62 (53.4%)	
	4	7 (77.77%)	2 (22.22%)	9 (7.8%)	
qSOFA score	1	13 (28.89%)	32 (71.11%)	45 (38.8%)	0.001*
	2	28 (50.90%)	27 (49.09%)	55 (47.4%)	
	3	13 (81.25%)	3 (18.75%)	16 (13.8%)	

Abbreviations: *Significant at 5% level of significance; qSOFA = Quick Sequential Organ Failure Assessment; SOFA = Sequential Organ Failure Assessment Score; SIRS = Systemic Inflammatory Response Syndrome

The median SOFA score was significantly higher in non-survivors, 8.5 (6, 12) compared to that in survivors 5 (3.25, 7), (p=0.001). A qSOFA score of 1 was seen in 45 (38.8%) patients, of which 13 (28.89%) died. A qSOFA score of ≥2 was seen in 71 (61.21%) patients, out of which 41 (57.75 %) died. A SIRS score of 1 was seen in nine (7.75%) patients, out of which 1 (11.11%) died. A SIRS score of ≥2 was observed in 107 (92.24%) patients, out of which 54 (50.47%) died. In comparison to the survivors, the non-survivor group had a significantly higher proportion of patients with SIRS criteria scores ≥2 (p=0.04) and qSOFA scores ≥2 (p=0.001). This implies that higher SOFA, qSOFA, and SIRS criteria scores were significantly associated with increased mortality.

**Figure 1:**
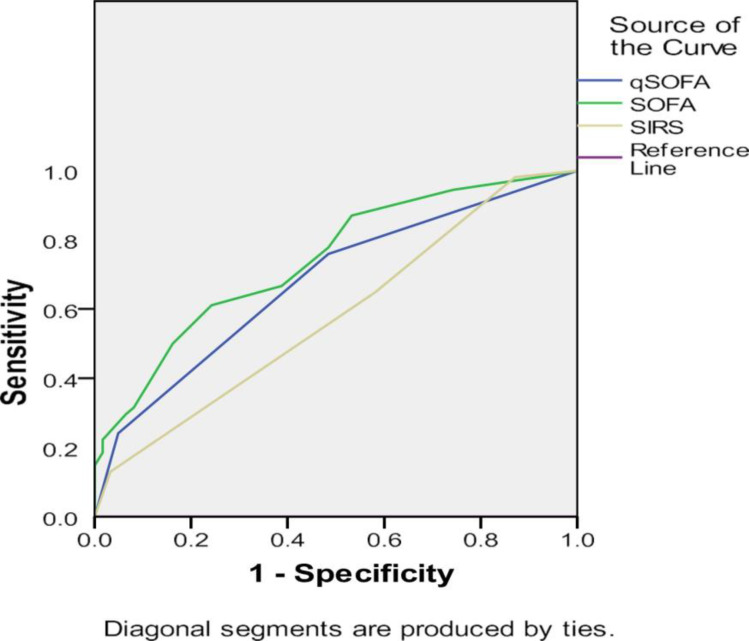
ROC for the three scores Figure legends ROC = Receiver operating curve ROC has been presented for qSOFA, SOFA, and SIRS.

Figure 1 presents the ROC for the three scores, and Table 4 shows the AUROC for the three scores. The AUROC for qSOFA ≥2 was 0.678 (p=0.001). This was comparable to the Table 1SOFA score for which AUROC was 0.741 (p=0.000), whereas AUROC for SIRS ≥2 was 0.580, and it was not statistically significant (p=0.139). This implies that a qSOFA score of >2 is comparable to SOFA and is better than a SIRS score of >2 for predicting mortality.

**Table 4:** AUROC for the three scores

Parameter	AUROC	p-value	Asymptotic 95% confidence interval	
			Lower bound	Upper bound
qSOFA score	0.678	0.001*	0.580	0.775
SOFA score	0.741	0.000*	0.652	0.831
SIRS criteria	0.580	0.139	0.476	0.684

Abbreviations: *Significant at 5% level of significance; AUROC = Area under receiver operating curve; qSOFA = Quick Sequential Organ Failure Assessment; SOFA = Sequential Organ Failure Assessment Score; SIRS = Systemic Inflammatory Response Syndrome

## Discussion

Sepsis is one of the leading causes for mortality worldwide, which can be reduced by early recognition and appropriate treatment.^1^,^2^ Hence, this study was conducted to compare SOFA score, qSOFA score, and SIRS criteria as predictors of mortality in patients with sepsis.

Similar age distribution was seen by Kovach et al, with a median age of 52 years and 62 years among survivors and non-survivors, respectively.^13^ Bronchopneumonia or other respiratory tract infections were identified as the most common cause of sepsis by Freund et al (43%) and Park et al (43.7%), mirroring the current research.^9^,^14^

The clinical and laboratory parameters observed were also in concordance with other researchers, e.g., tachycardia was noted in all patients by Freund et al (p=0.02) and Kim et al (p=0.032), who also found that non-survivors showed a significantly higher median PR (p=0.02, p=0.032) and a significantly lower median SBP (p<0.001, p=0.045) compared to the survivors.^9^,^15^ However, the difference between DBP was not significant (p=0.085) in the study of Kim et al, as was the difference in temperature, total leucocyte count, and platelet count (p=0.66) in the study by Boillat-Blanco et al.15,16 Similar to the present study, E. coli was the most common organism isolated in sputum and urine cultures by Ren et al and Manhal et al.^17^,^18^

The mortality in our study was 46.6 %, which is higher than previous studies. Probable reasons for this could be: Since our hospital is a tertiary care centre, patients have presented late to the hospital either in septic shock or in respiratory failure. 52.59% of the patients in our study presented to our hospital in septic shock and 69 (59.5%) patients required mechanical ventilation, and mortality occurred in 49 (71.01%) patients requiring mechanical ventilation.

The median SOFA scores also found resonance in the works of Khwannimit et al (12 in non-survivors and 6 in survivors).^19^ The AUROC for qSOFA ≥2 was 0.678 (p=0.001). This was comparable to SOFA score for which AUROC was 0.741 (p=0.000), whereas AUROC for SIRS ≥2 was 0.580, which was not statistically significant (p=0.139). In accordance with the present study, Raith et al and Kim et al observed that the AUROC (for predicting mortality) was 0.607 and 0.627 for qSOFA, respectively, 0.589 and 0.540 for SIRS, respectively, and 0.753 and 0.687 for SOFA, respectively, at 95% confidence interval.^6^,^15^ Maitra et al also found qSOFA (AU-ROC-0.74, 95% CI, 0.70–0.78) to be significantly better than SIRS criteria (AUROC-0.67, 95% CI, 0.63–0.71) in predicting mortality, similar to the present study.^20^ In contrast, Siddiqui et al found SIRS criteria (AUROC-0.7073, p=0.001) to be better than the qSOFA score (AU-ROC-0.6875) in predicting mortality.^7^

SOFA was found to be the best predictor of mortality in sepsis patients and qSOFA showed nearly comparable results, as confirmed by Raith et al and Freund et al.^6^,^9^

Early detection and initiation of antibiotics and the 1-hour sepsis bundle have been shown to reduce mortality in sepsis. There was a recent change in the definition of sepsis, which requires an acute change in SOFA score by 2 or more points. This can be cumbersome as the SOFA score requires a variety of laboratory parameters. A new Quick SOFA score was introduced, which is entirely clinical and can be done in under a minute and repeated at varying intervals. The need for determining the best scoring system for triaging sepsis patients arises from the lack of rapid diagnostic tests to accurately identify those at the greatest risk of mortality. Moreover, diagnostic tests for sepsis, such as culture and sensitivity testing, take a minimum of 48 hours to become positive, which delays the specific antibiotic administration, resulting in poorer outcomes. Thus, such a prognostic scoring system could supplement the physicians' clinical judgment to initiate aggressive treatment and save time and cost for the patient.^21^

However, this study has certain limitations in being single-centred with limited sample size. These can be overcome by multicentric long-term prospective studies with larger sample size.

## Conclusion

A qSOFA score of ≥ 2 is comparable to SOFA and is better than SIRS score ≥2 for predicting mortality.
